# Elevated Surgical Pleth Index at the End of Surgery Is Associated with Postoperative Moderate-to-Severe Pain: A Systematic Review and Meta-Analysis

**DOI:** 10.3390/diagnostics12092167

**Published:** 2022-09-06

**Authors:** Kuo-Chuan Hung, Yen-Ta Huang, Jinn-Rung Kuo, Chih-Wei Hsu, Ming Yew, Jen-Yin Chen, Ming-Chung Lin, I-Wen Chen, Cheuk-Kwan Sun

**Affiliations:** 1 Department of Anesthesiology, Chi Mei Medical Center, Tainan City 71004, Taiwan; 2 Department of Hospital and Health Care Administration, College of Recreation and Health Management, Chia Nan University of Pharmacy and Science, Tainan City 71710, Taiwan; 3 Department of Surgery, National Cheng Kung University Hospital, College of Medicine, National Cheng Kung University, Tainan City 70101, Taiwan; 4 Department of Neurosurgery, Chi Mei Medical Center, Tainan City 71004, Taiwan; 5 Department of Medical Research, Chi Mei Medical Center, Tainan City 71004, Taiwan; 6 Department of Psychiatry, Kaohsiung Chang Gung Memorial Hospital and Chang Gung University College of Medicine, Kaohsiung City 83301, Taiwan; 7 Department of Anesthesiology, Chi Mei Medical Center, Liouying, Tainan City 73657, Taiwan; 8 Department of Emergency Medicine, E-Da Hospital, Kaohsiung City 82445, Taiwan; 9 College of Medicine, I-Shou University, Kaohsiung City 84001, Taiwan

**Keywords:** surgical pleth index, pain, postanesthesia care unit, summary receiver operating characteristic, meta-analysis

## Abstract

Despite acceptance of the surgical pleth index (SPI) for monitoring the intraoperative balance between noxious stimulation and anti-nociception under general anesthesia, its efficacy for predicting postoperative moderate-to-severe pain remains unclear. We searched electronic databases (e.g., Google Scholar, MEDLINE, Cochrane Library, and EMBASE) to identify articles focusing on associations of SPI at the end of surgery with immediate moderate-to-severe pain in the postanesthesia care unit from inception to 7 July 2022. A total of six observational studies involving 756 adults published between 2016 and 2020 were eligible for quantitative syntheses. Pooled results revealed higher values of SPI in patients with moderate-to-severe pain than those without (mean difference: 7.82, 95% CI: 3.69 to 11.95, *p* = 0.002, I^2^ = 46%). In addition, an elevated SPI at the end of surgery was able to predict moderate-to-severe pain with a sensitivity of 0.71 (95% confidence interval (CI): 0.65–0.77; I^2^ = 29.01%) and a specificity of 0.58 (95% CI: 0.39–0.74; I^2^ = 79.31%). The overall accuracy based on the summary receiver operating characteristic (sROC) curve was 0.72. In conclusion, this meta-analysis highlighted the feasibility of the surgical pleth index to predict postoperative moderate-to-severe pain immediately after surgery. Our results from a limited number of studies warrant further investigations for verification.

## 1. Introduction

Nociception is defined as neural encoding of actual or impending tissue damage (e.g., noxious stimulation), while pain is the subjective experience pertinent to impending or actual harm [1]. Despite the current in-depth understanding of pain mechanisms and advances in pain management strategies, postoperative pain control remains challenging for physicians [2,3]. A previous meta-analysis involving 53,362 patients identified smoking, younger age, female sex, a history of depressive and anxiety symptoms, sleep difficulties, higher body mass index, the presence of preoperative pain, and the use of preoperative analgesia as significant preoperative predictors of poor postoperative pain control [4]. Unrelieved postoperative pain not only delays recovery [5], as well as increasing the risks of morbidity and mortality (e.g., myocardial infarction and pneumonia) [6,7], but also extends the length of hospital stays [8], increases patient dissatisfaction with medical care [9], and even predisposes to the development of chronic pain [10,11]. In addition to immediate postoperative pain, previous studies reported that patients often experienced postoperative pain in ward [12,13] and even after discharge from hospital [2]. Inadequate pain management has been attributed, at least partly, to the discrepancy between a patient’s subjective sensation of pain and the objective assessment of pain severity conducted by medical staff [12,13]. Cognitive, visual, or hearing impairment also contributes to difficulty in the accurate assessment of pain in some patient subgroups (e.g., the elderly) [14]. To optimize pain management, reliable objective measures for pain assessment or prediction [15,16] are necessary.

The surgical pleth index (SPI, GE Healthcare, Helsinki, Finland), which is derived from the normalized heart beat interval (HBI_norm_) and pulse photoplethysmographic amplitude (PPGA_norm_) from pulse oximetry measurements, has been proposed as a measure of the balance between noxious stimulation and anti-nociception during surgery under general anesthesia [17,18,19]. Mathematically, SPI = 100 − (0.33*HBInorm + 0.67*PPGAnorm) [17]. Although pooled evidence from a previous meta-analysis demonstrated a significant reduction in opioid consumption associated with the use of SPI monitoring compared to that with clinical signs intraoperatively, that study showed no significant effect of three nociception monitoring approaches (i.e., SPI, analgesia nociception index, and pupillometry-guided intraoperative analgesia) on the severity of postoperative pain and opioid requirement [20]. In the postoperative setting, SPI before arousal at the end of surgery has been shown to be higher in patients with postoperative moderate-to-severe pain than in those with no or mild pain in the postanesthesia care unit (PACU) [21,22], highlighting the significance of SPI in predicting postoperative pain immediately after surgery. Nevertheless, the efficacy of SPI for postoperative pain prediction based on the receiver operating characteristic (ROC) curve varied among studies [23,24,25]. The current meta-analysis aims to evaluate the diagnostic efficacy of SPI at the end of surgery in the prediction of postoperative moderate-to-severe pain.

## 2. Materials and Methods

### 2.1. Protocol, Literature Search, and Study Selection

The protocol of the present meta-analysis, which was previously registered on PROSPERO with the registration number CRD42022344376, followed the statement of the Preferred Reporting Items for Systematic Reviews and Meta-Analysis (PRISMA 2020) (Supplementary Table S1).

We searched electronic databases, including Google Scholar, MEDLINE, Cochrane Library, and EMBASE, to identify articles focusing on the associations of SPI at the end of surgery with immediate moderate-to-severe pain in the PACU from inception to 7 July 2022. These databases were independently searched by two reviewers using the following keywords: (“Surgical pleth index” or “surgical stress index”) and (“Postoperative pain” or “Visual analog scale” or “Numeric rating scale” or “Post-surgical pain”). There were no limitations on gender distribution and language. We also screened for additional records through examining the reference lists of the acquired articles. The search strategies are available in Supplementary Table S2.

Articles were considered eligible if the following PICOS criteria were met: (1) population: adults undergoing operations under general anesthesia being sent to PACU for postoperative care regardless of the type of surgery; (2) intervention: available SPI values before arousal at the end of surgery; (3) comparator: none; (4) outcomes: information about postoperative pain severity measured with reliable methods (e.g., numeric rating scale (NRS)) in the PACU; and (5) studies: observational studies with data on true-positive (TP), false-positive (FP), false-negative (FN), and true-negative (TN) values. For those without relevant information, adequate data for relevant calculation (i.e., case number, sensitivity, specificity, negative predictive value (NPV), and positive predictive value (PPV)) were deemed necessary. Discrepancy in opinion between the two reviewers regarding study eligibility was resolved through discussion with a third reviewer.

### 2.2. Methodologic Quality Assessment

The quality of the enrolled studies was evaluated by two independent reviewers with the Quality Assessment of Diagnostic Accuracy Studies 2 (QUADAS-2) that comprised four domains, namely, patient selection, index test, reference standard, and flow and timing. While all four domains were used for risk of bias assessment, the first three were examined in terms of concerns regarding applicability [26]. Disagreement regarding quality assessment results was settled through arbitration involving a third reviewer. Review Manager version 5.3 (Copenhagen: The Nordic Cochrane Centre, The Cochrane Collaboration, 2014) was used for a diagrammatic presentation of the QUADAS-2 results.

### 2.3. Data Extraction and Definition

The following items were extracted by two independent reviewers from the studies: (1) first author’s name (publication year), (2) gender distribution, (3) total patient number, (4) values of SPI at the end of surgery, (5) incidence of moderate-to-severe pain, (6) patient age, (7) area under the curve (AUC), (8) cut-off values of SPI for prediction of moderate-to-severe pain, (9) sensitivity/specificity or PPV/NPV, and (10) country. The definition of moderate-to-severe pain was based on that of individual studies. Inconsistent data were resolved by consensus-based discussion.

### 2.4. Data Synthesis and Analysis

The statistical analysis was previously reported in our studies [27,28]. The conversion of sensitivity/specificity or PPV/NPV into TP/TN/FP/FN was conducted with Review Manager version 5.3. Using the MIDAS command in Stata 15 (StataCorp LLC., College Station, TX, USA), the pooled estimates of sensitivity, specificity, positive and negative likelihood ratios (LR (+) and LR (-)), and the corresponding 95% confidence interval (CI) were computed based on the bivariate model [29]. I^2^ statistics was used to evaluate statistical heterogeneity across the included studies based on a random effects model. The overall accuracy was assessed with the AUC derived from the summary receiver operating characteristic (sROC) curve.

Posttest probability was evaluated with Fagan’s nomogram plot analysis according to pretest probability and LR (+)/LR (-). Fagan’s nomogram plot, a graphic tool, consists of a line that is drawn from pretest probability to pretest probability through LR (+) and LR (-) in the middle of the graph with the intersection point being set as the new estimated probability. It was used to estimate the improvement in disease detection using the diagnostic approach in question as reflected by an increase in posttest probability or vice-versa for the prediction of the absence of a disease. Potential publication bias was detected with Deeks’ funnel plot asymmetry test with statistical significance set at *p* < 0.05. The MIDAS command in Stata 15.2.4. was adopted for graphic presentations of Forest plots on pooled sensitivity and specificity, sROC curve, Deeks’ funnel plot, and Fagan’s nomogram plot.

## 3. Results

### 3.1. Characteristics of the Studies Included

**Figure 1:**
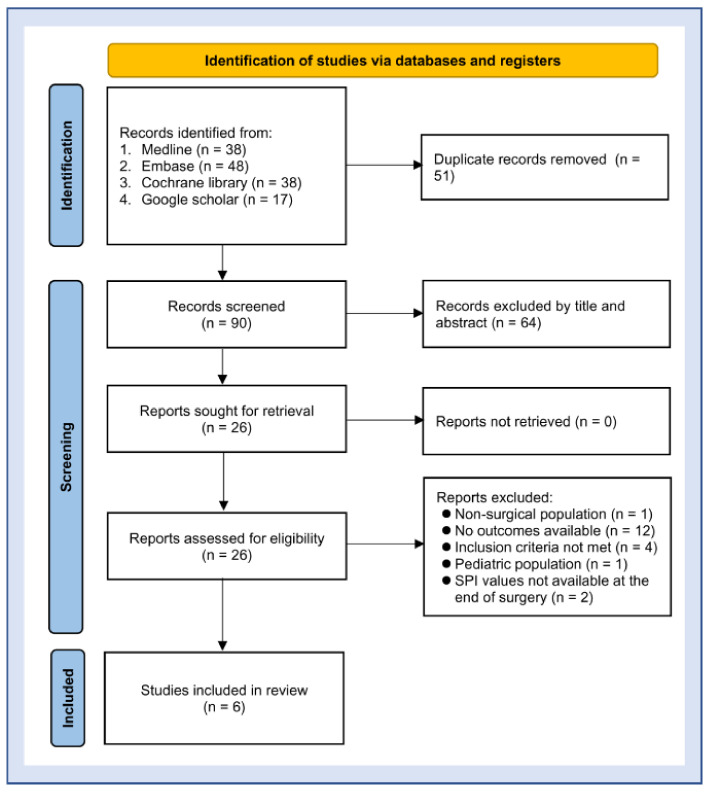
Flow chart for exclusion and inclusion of studies [21,23,24,25,30,31].

Figure 1 is the PRISMA diagram flowchart of the study selection process. Of the 141 articles initially identified through database screens (PubMed, 38; Embase, 48; Cochrane CENTRAL, 38; and Google Scholar, 17), 90 were examined by independent reviewers following the removal of duplicate records. Based on the inclusion and exclusion criteria, 64 records were further excluded by title and abstract. Among the remaining 26 studies retrieved for full-text review, 20 were removed for failing to meet the inclusion criteria (Supplementary Table S3). Finally, six observational studies involving adult surgical patients were included for the current meta-analysis [21,23,24,25,30,31].

**Figure 2:**
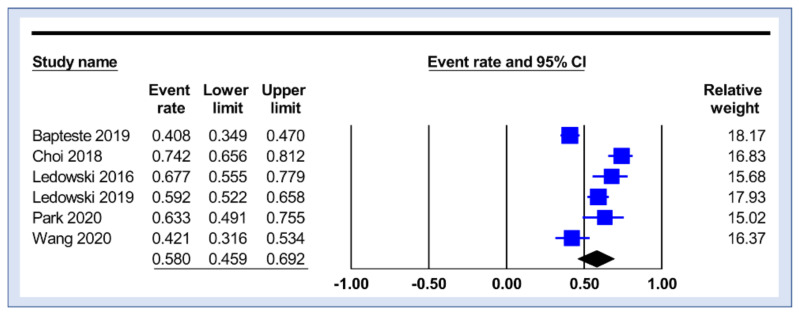
Pooled incidence of moderate-to-severe pain among studies [21,23,24,25,30,31].

The study characteristics are presented in Table 1. All studies were conducted prospectively and published between 2016 and 2020. A total of 756 participants undergoing surgeries were recruited with an age range of 43–68 years. All studies excluded participants who used medications with potential interactions with the sympathovagal balance [21,23,24,25,30,31]. Frequency of the female gender ranged between 22.4% and 100%. There was great variation in sample size across the studies, with a range of 49–250 participants. The incidence of moderate-to-severe pain in the PACU was between 40% and 74%, with a pooled incidence of 58% (Figure 2). Three studies reported an AUC of more than 0.7 (range: 0.703–0.8419) [21,23,25], while three studies described an AUC between 0.601 and 0.687 [24,30,31].

### 3.2. Quality of the Enrolled Studies

**Figure 3:**
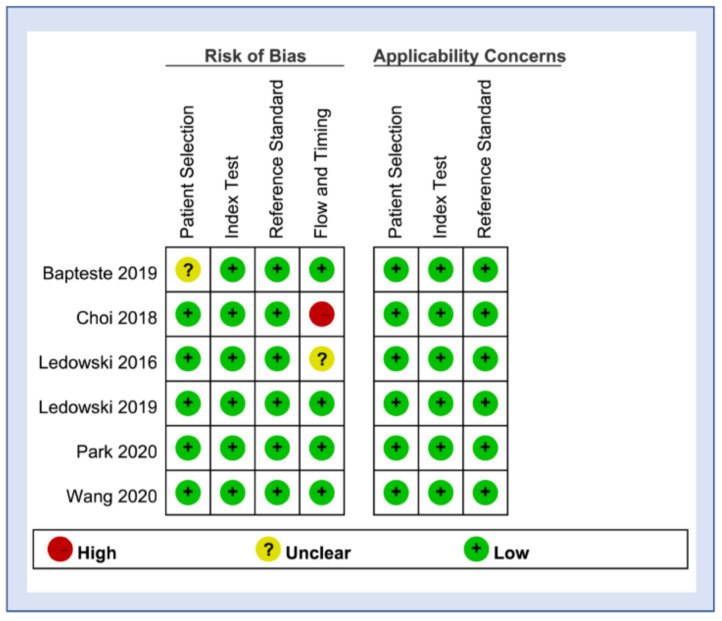
Risks of bias assessed according to the Quality Assessment of Diagnostic Accuracy Studies 2 (QUADAS-2) [21,23,24,25,30,31].

The methodological quality of the included studies evaluated with QUADAS-2 is shown in Figure 3, where the risks of bias and applicability concerns are also presented. In terms of “avoid inappropriate exclusion” in QUADAS-2, one study that did not clearly mention the exclusion criteria for patient selection was rated as having an unclear risk of bias in the aspect of patient selection [30]. Regarding the risk of bias in the domain of flow and timing, 25.5% [23] and 7.7% [21] of patients were excluded from analysis in two studies with their risk of bias in this domain being judged as high [23] and unclear [21], respectively. Regarding applicability, all studies were considered to have a low risk of bias in the domains of patient selection, index test, and reference standard.

### 3.3. Data Analysis

#### 3.3.1. SPI in Patients with or without Moderate-to-Severe Pain

**Figure 4:**
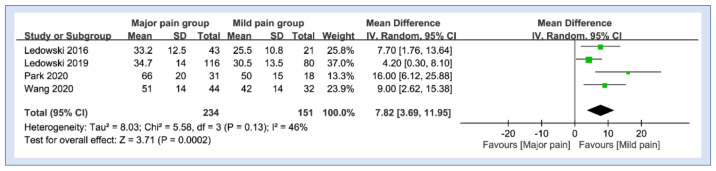
Forest plot comparing values of surgical pleth index (SPI) between patients with or without moderate-to-severe pain. IV: inverse variance; CI, confidence interval [21,24,25,31].

Details from four studies were available for analysis of the difference in SPI at the end of surgery in patients with or without moderate-to-severe pain in the PACU [21,24,25,31]. Merged results revealed higher values of SPI in patients with moderate-to-severe pain compared to those without (MD: 7.82, 95% CI: 3.69 to 11.95, *p* = 0.002, I^2^ = 46%) (Figure 4).

#### 3.3.2. Sensitivity/Specificity, sROC, and Publication Bias

**Figure 5:**
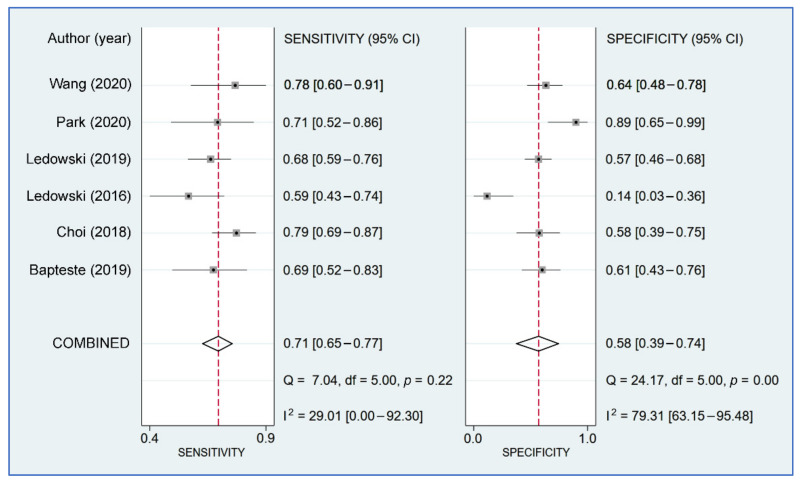
Forest plots comparing sensitivity/specificity and summarizing the pooled effects of the included studies [21,23,24,25,30,31].

**Figure 6:**
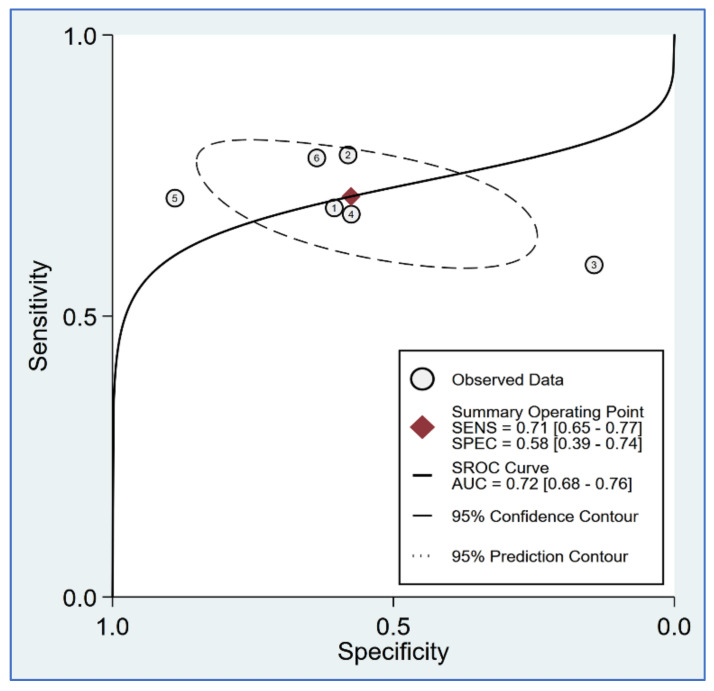
Hierarchical summary receiver operating characteristic (hsROC) curves of using surgical pleth index (SPI) for the prediction of moderate-to-severe pain in surgical patients [21,23,24,25,30,31]. SROC: summary receiver operating characteristic; SENS: sensitivity; SPEC: specificity; AUC: area under the curve.

**Figure 7:**
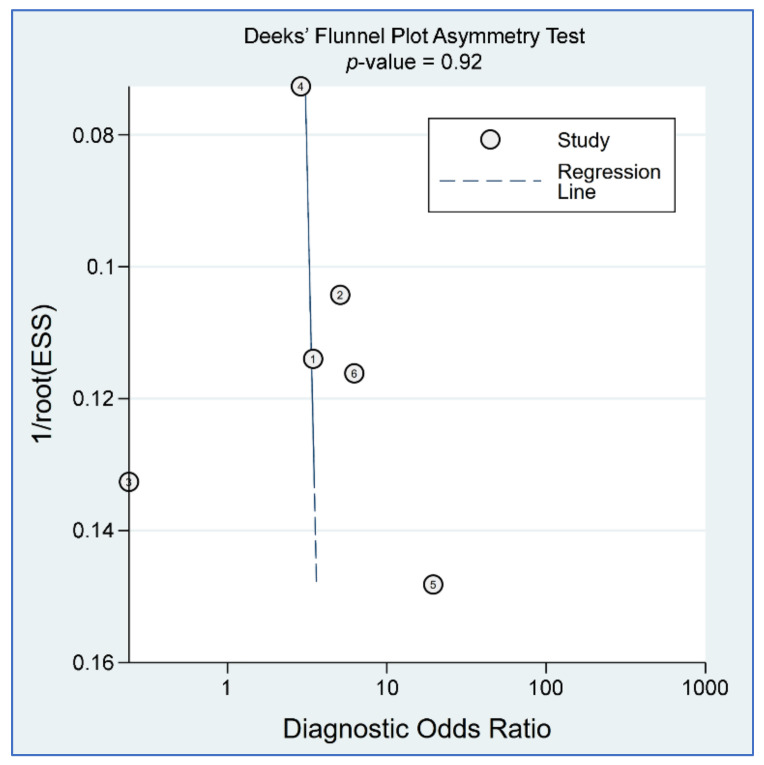
Deeks’ funnel plot asymmetry test for publication bias assessment across the included studies [21,23,24,25,30,31].

The sensitivity in individual studies ranged from 0.592 to 0.7816, while the specificity varied between 0.145 and 0.88 among the six included studies. Pooled sensitivity and specificity were 0.71 (95% CI = 0.65–0.77; I^2^ = 29.01%) and 0.58 (95% CI = 0.39–0.74; I^2^ = 79.31%), respectively (Figure 5). Linear regression for sROC derived from mathematical computation of the data on true and false positivity (1-specificity) of an individual study (Figure 6) revealed an AUC of 0.72 (95% CI: 0.68–0.76). A *p*-value of 0.92 from Deeks’ linear regression test indicated non-significant publication bias (Figure 7).

#### 3.3.3. Fagan’s Nomogram Plot Analysis

**Figure 8:**
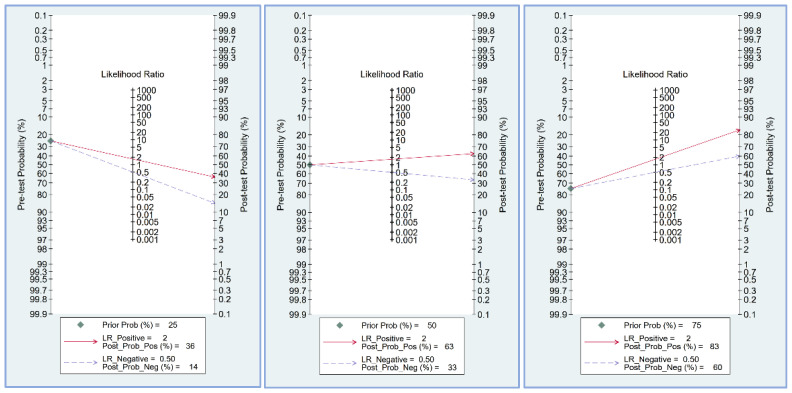
Fagan’s nomogram plot for evaluating clinical utility of surgical pleth index (SPI) for postoperative moderate-to-severe pain prediction in surgical patients [21,23,24,25,30,31]. LR: Likelihood ratio; Prob: probability; Pos: positive; Neg: negative.

Our analysis showed that pooled LR (+) and LR (-) were 2.00 and 0.5, respectively (Figure 8). In our study, when the pretest probabilities were 25% (Figure 8, left panel), 50% (Figure 8, middle panel), and 75% (Figure 8, right panel) based on the physician’s clinical judgment, the posttest probabilities referring to LR (+) were 36%, 63%, and 83%, respectively, and the posttest probabilities pertinent to LR (-) were 14%, 33%, and 60%, respectively (Figure 8). In summary, the prediction of moderate-to-severe pain was elevated after the use of SPI.

## 4. Discussion

Accurate predictions of moderate-to-severe pain in patients immediately after surgery enable the implementation of prophylactic pain management strategies for improving the quality of recovery and subsequent care. Our study aimed to explore the efficacy of SPI for predicting postoperative moderate-to-severe pain in adults. We found that the values of SPI before arousal at the end of surgery were higher among surgical patients with moderate-to-severe pain compared to those without in the PACU (i.e., mean in difference: 7.82). The pooled sensitivity, specificity, and AUC were 0.71, 0.58, and 0.72 respectively, suggesting that SPI may be a useful predictor of subjective pain perception in adult patients undergoing general anesthesia.

In a large-scale retrospective study involving 92,136 surgical patients focusing on critical incidents including moderate-to-severe pain (i.e., NRS > 3) and immediate complications in the PACU, the incidence of moderate-to-severe pain was 26.9%, which accounted for almost one-third of all patients in that study [32]. Moreover, another study reported moderate-to-extreme pain in up to 64% of inpatients before hospital discharge [2]. In the current meta-analysis, the pooled incidence of moderate-to-severe pain was 58% in the PACU, further highlighting inadequate analgesia during the early postoperative period and the need for improving analgesic strategies in clinical practice. The relatively high prevalence of moderate-to-severe pain in the included patients may be attributed to the timing of pain assessment, which was immediately following anesthesia in the recovery room before any analgesic intervention. Regarding the use of predictors of postoperative moderate-to-severe pain, one study on orthopedic patients identified preoperative smoking and the existence of severe systemic disease as significant predictors of severe postoperative pain during the immediate postoperative period [33]; while the odds of severe postoperative pain were 2.42-fold higher in smokers than in non-smokers, the probability of severe pain was 4.27-fold lower in patients with severe systemic disease compared to those without [33]. Another study on orthopedic trauma patients demonstrated that female gender and prior post-injury surgery were associated with severe acute pain in the PACU [34]. These findings suggest that the predictors of moderate-to-severe postoperative pain may vary across clinical settings and populations. Accordingly, a simple index, which is applicable in different settings and patient populations that can increase the accuracy of moderate-to-severe pain prediction may improve the quality of postoperative care.

SPI, which is calculated based on the pulse photoplethysmographic amplitude and heart rate interval derived from pulse oximetry measurements, has been clinically used for intraoperative surveillance of the balance between nociceptive stimuli and antinociceptive drug effects [17]. In common with SPI, two other pain assessment approaches, namely, the analgesia nociception index and the nociception level (NoL) index, are also derived from physiological parameters. While ANI utilizes heart rate variability (HRV) for computation, NoL is based on photoplethysmography, HRV, skin conductance, and temperature [35]. Both ANI and NoL have been reported to be superior to conventional physiological monitoring using heart rate and blood pressure for evaluating intraoperative pain severity [35]. Several studies have reported that the efficacy of SPI for monitoring the nociception–antinociception balance may be influenced by several factors, such as the age of participants, anesthetic agents used, body mass index, and concomitant use of catecholamines (e.g., atropine) or vasoactive agents [18,36,37,38,39,40]. Nevertheless, two previous meta-analyses demonstrated an association of a reduction in opioid consumption with intraoperative SPI-guided analgesia compared to monitoring with conventional clinical parameters (e.g., vital signs) [20,41]. Furthermore, the use of SPI can be related to faster extubation after surgery compared with standard clinical practice [41]. Despite these encouraging results, the SPI-related reduction in opioid consumption was unable to reduce the severity of postoperative pain or the incidence of perioperative adverse events (e.g., nausea) [41].

Postoperative pain is a subjective entity encompassing the physiologic, cognitive, and emotional aspects of a patient’s experience that cannot be objectively evaluated. In addition, other patient-specific factors (e.g., gender, smoking, age, alcohol use, genetic variability, preoperative opioid use, surgical variables, and preoperative expectation of pain) may also influence postoperative pain intensity [42,43,44,45]. Accordingly, pain measurement at the PACU relies on the subjective reporting of the severity of pain. Nevertheless, pain assessment during the immediate postoperative period may be difficult, as the residual effects of intraoperative anesthetic adjuncts may influence the reporting of postoperative pain. Moreover, visual, hearing, or cognitive impairment may impede accurate pain assessment in certain patient subgroups (e.g., the aged) [14]. Therefore, the use of objective monitors to predict postoperative moderate-to-severe pain before patient arousal may improve the quality of patient care immediately after surgery.

Two previous studies failed to identify a significant association between SPI and the severity of postoperative pain among awake patients [46,47]. SPI, which evaluates pain severity based on sympathetic activity, may not be an ideal assessment for awake patients, not only because arousal is a strong sympathetic stimulus but also due to the questionable correlation between acute postoperative pain and the level of sympathetic activation [48]. In contrast, focusing on patients before arousal from general anesthesia, several studies have endorsed the ability of SPI to predict postoperative moderate-to-severe pain at the end of surgery [21,23,24,25,30,31]. For example, one study reported that SPI values acquired within the last 10 min of surgery could predict moderate-to-severe pain (i.e., NRS 4–10) during the first 15 min of recovery room admission with an AUC of 0.711 [21]. Consistently, another study demonstrated the usefulness of SPI for the prediction of moderate-severe pain (NRS ≥ 5) in patients before arousal from general anesthesia with an AUC of 0.84 [25]. Because of the difference in clinical setting and the definition of moderate-to-severe pain, as well as the limited number of patients in our included studies [21,23,24,25,30,31], the current meta-analysis attempted to pool evidence to examine the accuracy of this technique to guide clinical practice. Our results show a pooled sensitivity and specificity of 0.71 and 0.58, respectively, with an AUC of sROC being 0.72. Therefore, despite the high sensitivity of SPI, its relatively low specificity suggests that it should be complemented with other technological monitoring devices and/or clinical signs in anesthesia practice. Although our findings suggest the feasibility of applying this parameter in surgical patients to improve postoperative pain management, further studies are required for verification.

A previous study reported a potential impact of age on the ability of SPI to discriminate differences in pain intensity in children at the end of surgery [22]. In that study [22], the AUCs in younger (i.e., 2–3 years) and older children (i.e., 9–16 years) were 0.83 and 0.63, respectively, indicating a better predictive capacity of SPI for moderate-to-severe pain in younger children. Moreover, a previous review article demonstrated a consistent association between the intensity of pain evoked by a preoperative suprathreshold heat stimulus and the level of postoperative pain in female but not male patients [49], suggesting that the accuracy of pain prediction may be gender-dependent. Nevertheless, the existence of sex- and age-related differences in pain sensitivity and tolerance as previously reported [50,51] remains unknown. In addition, age- and gender-associated differences in heart rate variability [52] may affect the accuracy of SPI, which is derived from pulse amplitude and heartbeat intervals, in discriminating differences in pain intensity. Therefore, our findings should be further supported by well-controlled, large-scale studies.

Our results should be judiciously interpreted in the light of several limitations in the present study. First, genetic factors, type of surgery, patient characteristics (e.g., age, and body weight), anxiety, and other psychological stress were found to be predictors of postoperative pain and analgesic consumption [43,53]; however, the confounding effects of these factors were not evaluated in studies included in the current meta-analysis. Other confounders of SPI interpretation also include medical conditions (e.g., hypertension) and intravascular filling status of the patient [54], both of which may bias the accuracy of SPI. Second, the limited number of studies may impair the robustness of evidence from the current study. Third, all studies did not focus on a specific type of procedure; therefore, whether there was a higher diagnostic efficacy of SPI in patients receiving certain types of procedures remains unknown. Fourth, because all studies focused on pain assessment during the immediate postoperative period, we cannot exclude the influences of intraoperative sedatives and anesthetics on the results of postoperative pain score assessment. Finally, since the association of SPI with late postoperative pain (e.g., in the ward) was not investigated in any of our studies, further investigation is required to clarify this issue. Whether an elevated end-of-surgery SPI could serve as a possible new risk factor for postoperative pain intensity remains to be elucidated in further studies.

## 5. Conclusions

The results of the current study demonstrate the feasibility of using the surgical pleth index for predicting moderate-to-severe pain in the immediate postoperative period in the postanesthetic care unit. Further studies are required to investigate whether the incorporation of this monitor could improve the quality of acute postoperative care.

## Figures and Tables

**Table 1:** Study characteristics (*n* = 6).

	Bapteste (2019) [30]	Choi (2018) [23]	Ledowski (2016) [21]	Ledowski (2019) [24]	Park (2020) [25]	Wang (2021) [31]
Observational study	Yes	Yes	Yes	Yes	Yes	Yes
Patient population	Adult women	Age: 20–80 years	Adult patients	Age: 18–95 years	Age: 20–80 years	Adult patients
Age (years)	NA	54.4	43	44	68	58.3
Number of patients	250	120	65	196	49	76
Female (%)	100	40	70.8	42.9	30.6	22.4
Type of surgery	Gynecologic surgery	Elective surgery	General surgery, *n* = 48; other surgical specialties *n* = 17	General surgery, *n* = 77; other surgical specialties *n* = 119	Liver resection	Urological surgery
Risk factors for pain	Female gender	NA	Female gender	NA	Liver resection	NA
Exclusion criteria	a,b,c	a,b,c	a,b,c	a,b,c	b,c	a,b,c
Outcome parameters (Measure)	Pain severity (VAS)	Pain severity (NRS)	Pain severity (NRS)	Pain severity (NRS)	Pain severity (NRS)	Pain severity (NRS)
Definition	VAS > 3	NRS > 3	NRS > 3	NRS > 3	NRS ≥ 5	NRS > 3
Incidence (%)	40	74	68	59	63	42
Main findings: AUC/Cut-off value	0.657/53	0.703/43.7	0.711/30	0.601/29	0.8419/60	0.687/44

^a^ cardiac disease; ^b^ cardiac arrhythmia; ^c^ medication that affects autonomic function; AUC: area under the curve; NA: not available; NRS: numerical rating scale; VAS: visual analog scale; moderate-to-severe pain.

## Data Availability

Not applicable.

## Supplementary Materials

The following supporting information can be downloaded at: https://www.mdpi.com/article/10.3390/diagnostics12092167/s1, Table S1: PRISMA checklist; Table S2: Search strategies; Table S3: Excluded studies.
