# Supplementary material for: Elevated Surgical Pleth Index at the End of Surgery Is Associated with Postoperative Moderate-to-Severe Pain: A Systematic Review and Meta-Analysis
Source: Diagnostics (Basel). 2022 Sep 6;12(9):2167. doi: 10.3390/diagnostics12092167 (PMC9498235; doi:10.3390/diagnostics12092167)
Supplement: Supplementary file 1 [file diagnostics-12-02167-s001.zip › Supplementary Table S2.pdf]

Supplementary Table S2. Search strategies

Search strategies for Medline	
1	("Surgical pleth index" or "surgical stress index").mp.
2	("Postoperative pain" or "Visual analog scale" or "Numeric rating scale" or "Post-surgical pain").mp.
3	exp "Pain, Postoperative"/
4	1 and (2 or 3)
Search strategies for Embase	
1	("Surgical pleth index" or "surgical stress index"):ti,ab,kw,de
2	("Postoperative pain" or "Visual analog scale" or "Numeric rating scale" or "Post-surgical pain"):ti,ab,kw,de
3	"Postoperative pain"/exp
	1 and (2 or 3)
Search strategies for Cochrane CENTRAL	
1	("Surgical pleth index" or "surgical stress index"):ti,ab,kw
2	("Postoperative pain" or "Visual analog scale" or "Numeric rating scale" or "Post-surgical pain"):ti,ab,kw
3	[mh "Pain, Postoperative"]
	#1 and (#2 or #3)
Google scholar	
1	(Surgical pleth index) and (postoperative pain)
