# Supplementary material for: Elevated Surgical Pleth Index at the End of Surgery Is Associated with Postoperative Moderate-to-Severe Pain: A Systematic Review and Meta-Analysis
Source: Diagnostics (Basel). 2022 Sep 6;12(9):2167. doi: 10.3390/diagnostics12092167 (PMC9498235; doi:10.3390/diagnostics12092167)
Supplement: Supplementary file 1 [file diagnostics-12-02167-s001.zip › Supplementary Table S3.pdf]

Supplementary Table S3. Excluded studies

Reasons for exclusion	References
Non-surgical population	[1]
No outcomes available	[2-13]
Inclusion criteria not met	[14-17]
Pediatric population	[18]
SPI values not available at the end of surgery	[19,20]

References

[1] Funcke S, Sauerlaender S, Pinnschmidt HO, Saugel B, Bremer K, Reuter DA, et al. Validation of innovative techniques for monitoring nociception during general anesthesia: a clinical study using tetanic and intracutaneous electrical stimulation. Anesthesiology. 2017;127:272-83.

[2] Colombo R, Raimondi F, Corona A, Rivetti I, Pagani F, Della Porta V, et al. Comparison of the Surgical Pleth Index with autonomic nervous system modulation on cardiac activity during general anaesthesia: a randomised cross-over study. European Journal of Anaesthesiology | EJA. 2014;31:76-84.

[3] Guo J, Zhu W, Shi Q, Bao F, Xu J. Effect of surgical pleth index-guided analgesia versus conventional analgesia techniques on fentanyl consumption under multimodal analgesia in laparoscopic cholecystectomy: a prospective, randomized and controlled study. BMC Anesthesiology. 2021;21(1) (no pagination).

[4] Ho CN, Fu PH, Chen JY, Hung KC, Chang JH, Peng CK, et al. Heart rate variability and surgical pleth index under anesthesia in poor and normal sleepers. Journal of Clinical Monitoring and Computing. 2020;34(6):1311-9.

[5] Park JH, Lim BG, Kim H, Lee IO, Kong MH, Kim NS. Comparison of Surgical Pleth Index-guided Analgesia with Conventional Analgesia Practices in Children: A Randomized Controlled Trial. Anesthesiology. 2015;122:1280-7.

[6] Reddy KRM, Menon PA, Venkat S. Evaluating Nociception Indices Analgesia Nociception Index & Surgical Pleth Index in Elective Supratentorial Tumour Excision under General Anaesthesia-A Prospective Single Group Correlational Study. Anaesthesia and Intensive Care. 2020;48(1 SUPPL):4.

[7] Sun C-K, Chen I-W, Tsai I-T, Hung K-C. Association of age with accuracy of surgical pleth index to predict major postoperative pain. British Journal of Anaesthesia. 2020;124:e18-e9.

[8] Bergmann I, Göhner A, Crozier TA, Hesjedal B, Wiese CH, Popov AF, et al. Surgical pleth index-guided remifentanil administration reduces remifentanil and propofol consumption and shortens recovery times in outpatient anaesthesia. Br J Anaesth. 2013;110:622-8.

[9] Chen IW, Lin CM, Chang YJ, Chen JY, Wu ZF, Ho CN, et al. Association of surgical pleth index with late postoperative analgesic requirement: A retrospective study. J Clin Anesth. 2020;60:12-3.

[10] Colombo R, Raimondi F, Rech R, Castelli A, Fossali T, Marchi A, et al. Surgical Pleth Index guided analgesia blunts the intraoperative sympathetic response to laparoscopic cholecystectomy. Minerva Anestesiol. 2015;81:837-45.

[11] Gruenewald M, Harju J, Preckel B, Molnár Z, Yli-Hankala A, Rosskopf F,

et al. Comparison of adequacy of anaesthesia monitoring with standard clinical practice monitoring during routine general anaesthesia: An international, multicentre, single-blinded randomised controlled trial. Eur J Anaesthesiol. 2021;38:73-81.
[12] Jung K, Park MH, Kim DK, Kim BJ. Prediction of postoperative pain and opioid consumption using intraoperative surgical pleth index after surgical incision: an observational study. Journal of Pain Research. 2020;13:2815.
[13] Kim JH, Jwa EK, Choung Y, Yeon HJ, Kim SY, Kim E. Comparison of Pupillometry With Surgical Pleth Index Monitoring on Perioperative Opioid Consumption and Nociception During Propofol-Remifentanil Anesthesia: A Prospective Randomized Controlled Trial. Anesth Analg. 2020;131:1589-98.
[14] Ledowski T, Ang B, Schmarbeck T, Rhodes J. Monitoring of sympathetic tone to assess postoperative pain: skin conductance vs surgical stress index. Anaesthesia. 2009;64:727-31.
[15] Ledowski T, Sommerfield D, Slevin L, Conrad J, von Ungern-Sternberg B. Surgical pleth index: prediction of postoperative pain in children? BJA: British Journal of Anaesthesia. 2017;119:979-83.
[16] Lee JH, Choi BM, Jung YR, Lee YH, Bang JY, Noh GJ. Evaluation of Surgical Pleth Index and Analgesia Nociception Index as surrogate pain measures in conscious postoperative patients: an observational study. J Clin Monit Comput. 2020;34:1087-93.
[17] Thee C, Ilies C, Gruenewald M, Kleinschmidt A, Steinfath M, Bein B. Reliability of the surgical Pleth index for assessment of postoperative pain: a pilot study. European Journal of Anaesthesiology| EJA. 2015;32:44-8.
[18] Harju J, Kalliomäki ML, Leppikangas H, Kiviharju M, Yli-Hankala A. Surgical pleth index in children younger than 24 months of age: a randomized double-blinded trial. Br J Anaesth. 2016;117:358-64.
[19] Park C, Yang M-H, Choi B, Jeon B, Lee Y-H, Shin H, et al. Performance of the nasal photoplethysmographic index as an analgesic index during surgery under general anaesthesia. Scientific Reports. 2020a;10:1-7.
[20] Wang M, Wang X, Bao R, Zhu W-z, Bian J-j, Deng X-m, et al. Predictive value of the surgical pleth index for the hemodynamic responses to trachea intubation and skin incision. Journal of Clinical Monitoring and Computing. 2020;34:1303-9.
